# Synergistic AI-resident approach achieves superior diagnostic accuracy in tertiary ophthalmic care for glaucoma and retinal disease

**DOI:** 10.3389/fopht.2025.1581212

**Published:** 2025-05-19

**Authors:** Dalia Camacho-García-Formentí, Gabriela Baylón-Vázquez, Karen Arriozola-Rodríguez, Enrique Avalos-Ramirez, Curt Hartleben-Matkin, Hugo Valdez-Flores, Damaris Hodelin-Fuentes, Alejandro Noriega

**Affiliations:** ^1^ Tech & Intelligence Department, PROSPERiA, Mexico City, Mexico; ^2^ Glaucoma Department, Instituto de Oftalmología Fundación Conde de Valenciana Institución de Asistencia Privada (IAP), Mexico City, Mexico; ^3^ Retina Department, Hospital de Nuestra Señora de la Luz, Mexico City, Mexico; ^4^ Ophthalmology Department, Hospital General Dr. Juan Bruno Zayas Alfonso, Santiago de Cuba, Cuba

**Keywords:** glaucoma, cup-to-disc ratio, retinal disease, artificial intelligence, ophthalmology resident, tertiary care, fundus images

## Abstract

**Introduction:**

Artificial intelligence (AI) shows promise in ophthalmology, but its potential in tertiary care settings in Latin America remains understudied. We present a Mexican AI-powered screening tool and evaluate it against first-year ophthalmology residents in a tertiary care setting in Mexico City.

**Methods:**

We analyzed data from 435 adult patients undergoing their first ophthalmic evaluation using an AI-based platform and first-year ophthalmology residents. The platform employs an Inception V3–based multi-output classification model with 512 × 512 input resolution to capture small lesions when detecting retinal disease. To evaluate glaucoma suspects, the system uses U-Net models that segment the optic disc and cup to calculate cup-to-disc ratio (CDR) from their vertical heights. The AI and resident evaluations were compared with expert annotations for retinal disease, CDR measurements, and glaucoma suspect classification. In addition, we evaluated a synergistic approach combining AI and resident assessments.

**Results:**

For glaucoma suspect classification, AI outperformed residents in accuracy (88.6% vs. 82.9%, *p* = 0.016), sensitivity (63.0% vs. 50.0%, *p* = 0.116), and specificity (94.5% vs. 90.5%, *p* = 0.062). The synergistic approach achieved a higher sensitivity (80.4%) than ophthalmic residents alone or AI alone (*p <* 0.001). AI’s CDR estimates showed lower mean absolute error (0.056 vs. 0.105, *p <* 0.001) and higher correlation with expert measurements (*r* = 0.728 vs. *r* = 0.538). In the retinal disease assessment, AI demonstrated higher sensitivity (90.1% vs. 63.0% for medium/high risk, *p <* 0.001) and specificity (95.8% vs. 90.4%, *p <* 0.001). Furthermore, differences between AI and residents were statistically significant across all metrics. The synergistic approach achieved the highest sensitivity for retinal disease (92.6% for medium/high risk, 100% for high risk).

**Discussion:**

AI outperformed first-year residents in key ophthalmic assessments. The synergistic use of AI and resident assessments showed potential for optimizing diagnostic accuracy, highlighting the value of AI as a supportive tool in ophthalmic practice, especially for early career clinicians.

## 1 Introduction

The need for ophthalmic screenings has increased significantly due to the high prevalence of risk factors associated with ophthalmic diseases. Diabetes, hypertension, and increasing age are relevant risk factors for several conditions. These include glaucoma, diabetic retinopathy (DR), hypertensive retinopathy, diabetic macular edema (ME), age-related macular degeneration (AMD), and cataracts (1–5).

While periodic ophthalmic evaluations are recommended for these at-risk populations, Mexico faces a significant shortage of ophthalmologists, with only 4,213 registered in July 2024. Of these, 31.5% are concentrated in Mexico City (6). This scarcity makes comprehensive screening of all at-risk individuals unfeasible, considering that over 15 million Mexicans over 20 years old have diabetes, 40 million have hypertension, and the elderly population comprises over 18 million people (7–10).

To address the growing need for ophthalmic screenings worldwide, researchers have developed artificial intelligence (AI) models for retinal disease screenings using fundus images (11–18), as well as models that analyze the optic disc for glaucoma suspect classification (19–21).

Although most AI research in ophthalmology has been conducted in Asia, North America, and Europe (22), some AI systems and models have been validated in Latin America (13, 14, 17). For example, Arenas-Cavalli et al. have validated DART, a DR screening tool, on the Chilean health system (17), and González-Briceño et al. evaluated their models on primary care data from the Mexican Institute of Social Security (13).

These validations focused mainly on DR screening in primary care settings. To our knowledge, AI systems that identify a broader range of retinal diseases, as well as glaucoma suspects, have not been evaluated in Latin America. Furthermore, their potential in tertiary care settings remains unexplored, even though experienced ophthalmologists exhibit considerable variability in CDR estimation and retinal disease assessments (19, 23–25). Additionally, previous studies have demonstrated that AI can enhance ophthalmologists’ sensitivity in DR classification (26), and AI-based cup-to-disc ratio (CDR) measurements have surpassed the average expert (19).

AI can support early career clinicians in the learning process (27, 28); it could also be valuable in clinical practice, considering that first-year residents are often responsible for first-time consultations at ophthalmology hospitals in Mexico.

We present an AI screening tool trained on Mexican data to identify several retinal diseases, including DR, ME, AMD, pathological myopia (PM), and the presence of lesions associated with other retinal diseases. It also provides CDR estimation for glaucoma suspect classification, identifies possible media opacities, and offers explainability features.

We evaluated its performance during first-time consultations at a Mexican ophthalmology hospital and compared it against first-year ophthalmology residents. Our evaluation focused on CDR estimation, glaucoma suspect, and retinal disease classifications. We also compared cataract diagnoses by residents to media opacities detected by AI. Additionally, we explored a synergistic approach between AI and residents, highlighting the benefits of AI-human interaction in patient care.

Compared to previous research, our study presents a Latin American AI screening tool for multiple ophthalmic conditions and evaluates the specific use case of AI in an ophthalmology hospital in Latin America, comparing it against first-year ophthalmology residents who serve as the standard benchmark in Mexico for first-time ophthalmology consultations. Thus, we assess the possible improvements that could be achieved from implementing AI systems.

## 2 Materials and methods

To evaluate the screening tool, we conducted screenings on patients over 18 years old who underwent their first ophthalmic evaluation by a first-year ophthalmology resident. The study was carried out from 12 February to 14 March 2024 at Conde de Valenciana Centro, an ophthalmic institute in Mexico City.

This study adhered to the Declaration of Helsinki guidelines and was approved by Conde de Valenciana’s Ethics in Research Committee (CEI-2023/12/01), Biosecurity Committee (CB-0053-2023), and Research Committee (CI-053-2023).

For each patient, we collected their hospital medical record ID, personal information, relevant risk factors, and ophthalmic symptoms. A complete list of variables is provided in **Supplementary Table S1**.

Retinal fundus images were also required for screening. These were captured with a Horus 45° autofocus portable non-mydriatic fundus camera (Jedmed).

### 2.1 Data

A total of 464 patients were screened. Nine screenings were excluded due to registration errors in the AI platform, and six were removed because of missing images caused by camera malfunctions. Additionally, 14 patients were excluded due to empty medical records. Thus, 435 screenings were included in the final analysis.

A patient flow diagram illustrating the number of patients included in retinal disease evaluations and CDR assessments is provided in **Supplementary Figure S1**.

The average patient age was 59.1 years (*SD* = 15.7). Of the total sample, 34.0% were male and 66.0% were female, while 32.2% reported diabetes and 39.3% declared being previously diagnosed with hypertension.

### 2.2 Medical records

Patient medical records required for the study were identified by the medical record ID. The following information was extracted: CDR per eye, initial diagnosis assigned by the first-year residents, and whether the diagnosis was associated with glaucoma, retinal disease, cataracts, or another subspecialty.

### 2.3 Ground truth annotation for evaluation

A total of 1,013 fundus images were collected during data acquisition, of which 918 belonged to the 435 patients included in the analysis.

For CDR ground truth determination, three ophthalmologists annotated 861 images with visible optic disc, using the LinkedAI annotation platform (29). The ground truth CDR was calculated as the average CDR assigned by the three experts.

The Pearson’s correlation coefficients for CDR between ophthalmologists were as follows: 0.553 between ophthalmologists 1 and 2, 0.820 between ophthalmologists 1 and 2, and 0.650 between ophthalmologists 2 and 3. The correlation of each ophthalmologist’s annotations with the ground truth was 0.914, 0.802, and 0.934, respectively. These values indicate that ophthalmologist 2 exhibited higher variability compared to ophthalmologists 1 and 3.

**Figure 1:**
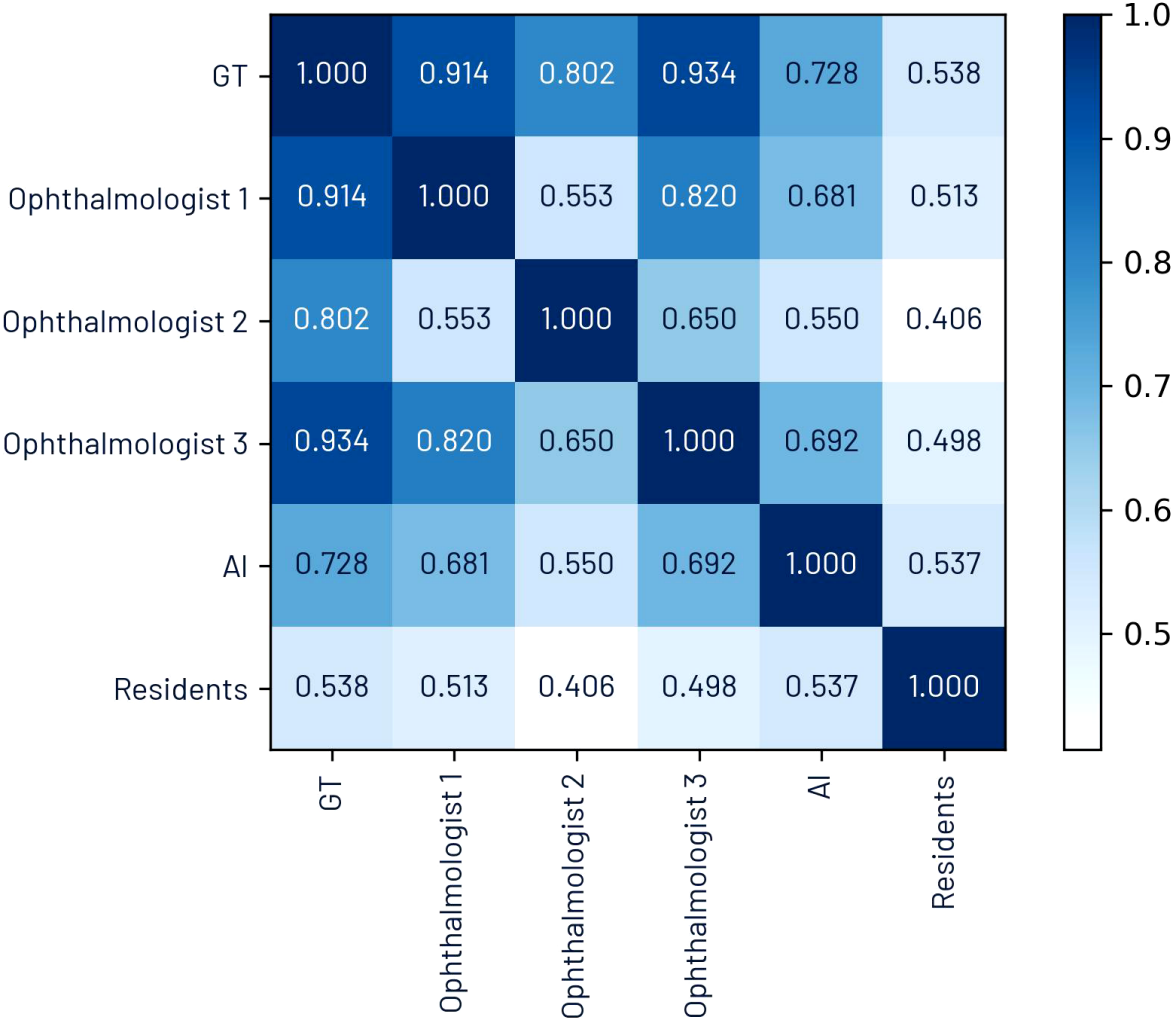
Pearson’s correlation coefficient matrix for CDR.

**Figure 1** presents a correlation matrix illustrating correlation coefficients between each ophthalmologist, the ground truth, the AI, and first-year residents.

Glaucoma suspect ground truth was defined as a CDR of 0.6 or higher or a CDR difference between both eyes exceeding 0.2. These criteria were based on Harizman et al’s definition of absence of glaucomatous optic neuropathy, considering CDR-related measurements (30).

For retinal disease ground truth determination, all 918 images were annotated by a retina specialist and an ophthalmic expert. In cases where the two experts disagreed, ground truth was established through consensus. An in-house built platform, Televal (31), was used for retinal annotation. A complete list of all annotated retinal findings is provided in **Supplementary Table S2**.

The criteria for prediagnosis were based on established clinical guidelines (4, 32–34), while the criteria for risk classification were informed by follow-up and treatment guidelines, including those from the Mexican Institute of Social Security (35) and the AMD Preferred Practice Pattern (4). Further details are provided in Section 4 of the **Supplementary Material**.

### 2.4 AI screening tool

The screenings for this study were conducted using retinIA (v3.3.1), an AI-based ophthalmic screening tool developed with Mexican data by PROSPERiA (36). Compared to other screening tools, such as EyeArt, DART, SELENA+, and Retinalyze, which primarily focus on detecting DR, AMD, or glaucoma, retinIA offers a more comprehensive assessment (11, 12, 17, 37–39). It identifies a broader range of retinal diseases, including DR, ME, AMD, and PM, as well as the presence of other retinal diseases and risk of visual loss. It also provides CDR estimation for glaucoma suspect classification, detection of possible media opacities and other ophthalmic conditions, and explainability features to enhance clinical interpretability.

This AI platform operates on cloud-based services, reducing computational demands on the user’s device. The minimum system requirements include a device with at least 4 GB of RAM, 200 MB of free storage, and a USB port or adapter; a supported web browser, including Google Chrome (v90+), Mozilla Firefox (v78+), Safari (v14+), or Edge (v91+); and an internet connection with at least 1.6 Mbps speed (or 3G for mobile connections).

#### 2.4.1 Retinal disease assessment

For retinal disease analysis, a multi-output convolutional neural network is used to determine image quality, laterality, presence of DR, ME, macular degeneration, pathological myopia, retinal lesions, and associated risks of vision loss.

**Figure 2:**
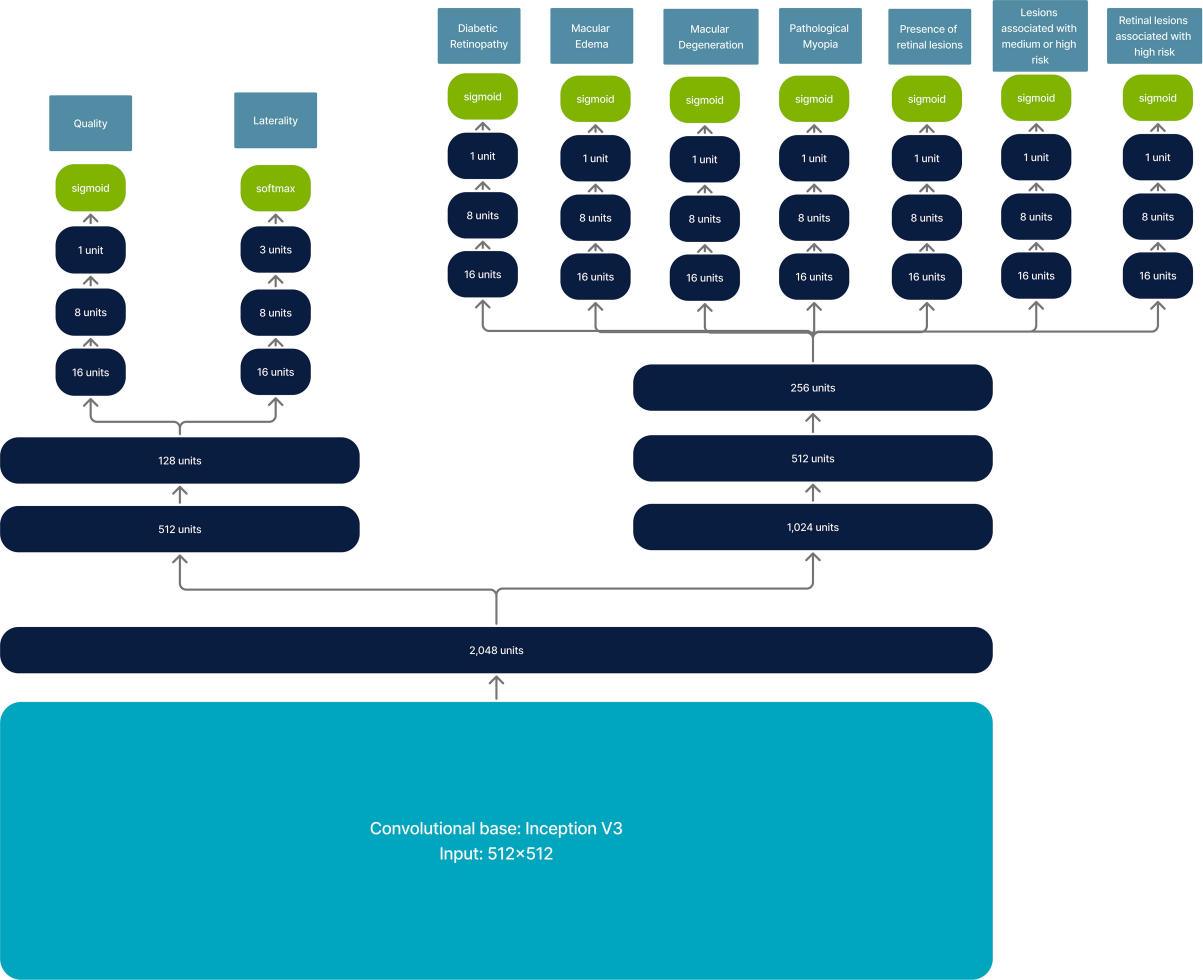
Model architecture for retinal image analysis. It has an input of 512 × 512 and returns multi-output classification.

The convolutional base corresponds to the Inception V3 architecture (40). This is followed by a dense layer of 2,048 units, which then splits into two branches: one for image quality and laterality and another for retinal disease and risk of vision loss analysis. Each output works independently for each classification task, resulting in architecture with multiple classification outputs. The complete architecture is shown in **Figure 2**.

As a pre-processing step, images are resized to 512 × 512. Typically, the input for Inception V3 is 299 × 299; however, we increased the input size to 512 × 512 to maintain the visibility of microaneurysms and small drusen after resizing. We also applied an image enhancement method based on Graham’s approach (41).

Other computer vision models, such as EfficientNet and Vision Transformers (ViT), have demonstrated better performance compared to Inception V3 in various tasks (42, 43). However, given the required input size of at least 512 × 512, using EfficientNet would require EfficientNet B6 (input size of 528 × 528), which has 76% more parameters than the selected model (45,256,650 vs. 25,706,075 parameters), significantly increasing computational requirements for both training and deployment.

Furthermore, pretrained ViT models such as RetFound (44) have input sizes of 224 × 224, which would result in the loss of small lesion details upon resizing. Additionally, implementing RetFound would increase the model size by a factor of 10.9 compared to the current Inception V3–based model (306,613,738 vs. 25,706,075 parameters). Moreover, internal validation has shown that Inception V3 performs well as a convolutional base.

The model was trained on Mexican data with a private dataset consisting of 104,216 fundus images collected between October 2020 and October 2023. Images were gathered from various screening locations, including optometry retail stores, primary care clinics, and cardiometabolic clinics. Collection sites spanned multiple Mexican states, including Mexico City, Mexico State, Chihuahua, Guanajuato, Nayarit, and Jalisco.

The images were captured using non-mydriatic fundus cameras, including the Horus 45° autofocus portable fundus camera from Jedmed (77.7%), DRS from Centervue (10.4%), and Visuscout 100 from Zeiss (2.2%). The remaining 9.7% of images were acquired using other camera models; however, these were only included in the training set. For internal validation and evaluation, only images from the Horus, DRS, and Zeiss cameras were used, with the majority of evaluation images (90.9%) coming from the Horus camera. While distribution across camera types is not balanced, the dataset represents data obtained in real-world screenings.

The average patient age, where this information was available, was 52 years, with 65% being female, 36% reporting diabetes, and 34% reporting hypertension.

Potential biases may arise from the dataset’s demographic characteristics. The average age in the dataset was 23 years older than the median age in Mexico in 2020 (29 years) (8). Additionally, the proportion of female patients was higher than that of the general population (51.2%). Consequently, diseases that are more prevalent in younger populations or in males may be underrepresented. Furthermore, since most of the data comes from individuals at risk of developing DR, signs associated with this condition may be overrepresented. It is also important to note that the dataset does not fully represent the general patient population, as tertiary care settings often exhibit greater variability in disease presentation compared to more homogeneous profiles typically observed in at-risk individuals in primary care.

A total of 91,884 images were used for training, 6,156 for validation, and 6,176 for internal testing. No data augmentation was applied during training.

All images were graded by either an ophthalmologist or a retina expert. The labeling approach involved annotating the presence of specific retinal lesions and applying logical rules based on disease classification guidelines to determine retinal disease presence and severity. The list of annotated lesions is presented in **Supplementary Table S2**, and the logical rules are detailed in **Supplementary Table S3**.

This AI tool also includes explainability features for retinal image analysis. These features provide a heat map for retinal anomalies, generated by combining GradCam (45) and SmoothGrad (46) methods.

**Figure 3:**
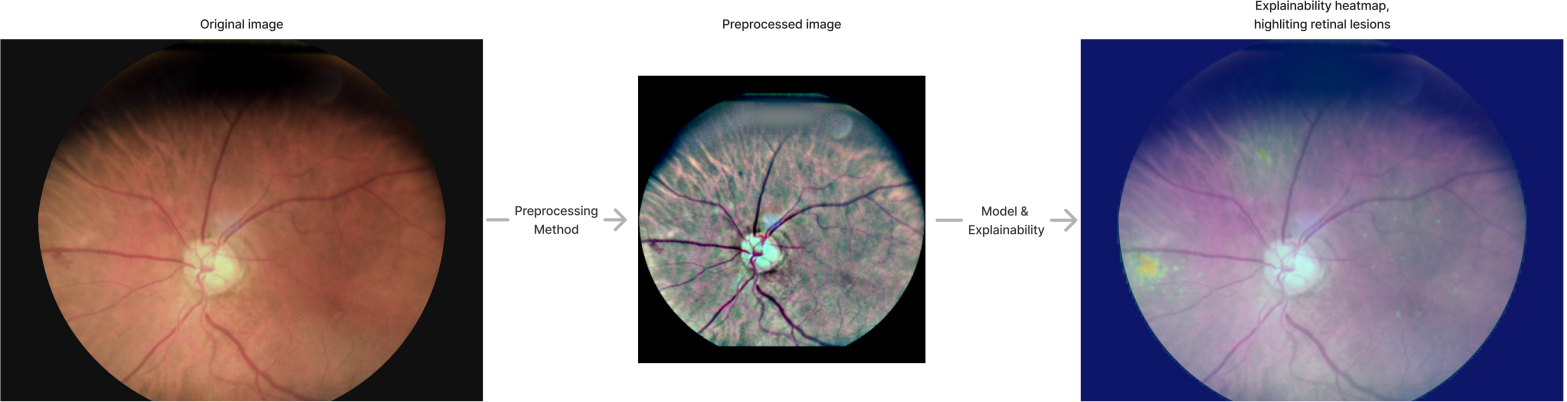
Pre-processing and explainability. The image indicates how the original image is pre-processed and shows explainability heatmap generated by the AI tool.

**Figure 3** illustrates an example of image preprocessing for model input and the corresponding explainability heatmap produced during post-processing.

#### 2.4.2 CDR estimation

CDR estimation involves several steps. First, images are resized to 256 × 256, and a U-Net architecture (47) is used to segment the optic disc and identify the region of interest (ROI) where the optic disc is present. The optic disc ROI is extracted and resized to 256 × 256. Image quality is then verified using a convolutional neural network based on Inception V3 with 256 × 256 input. If quality meets the criteria, a second U-Net is used to extract the optic cup. The segmented image is rescaled to the original ROI size, and the heights of the optic cup and optic disc are extracted to calculate CDR.

**Figure 4:**
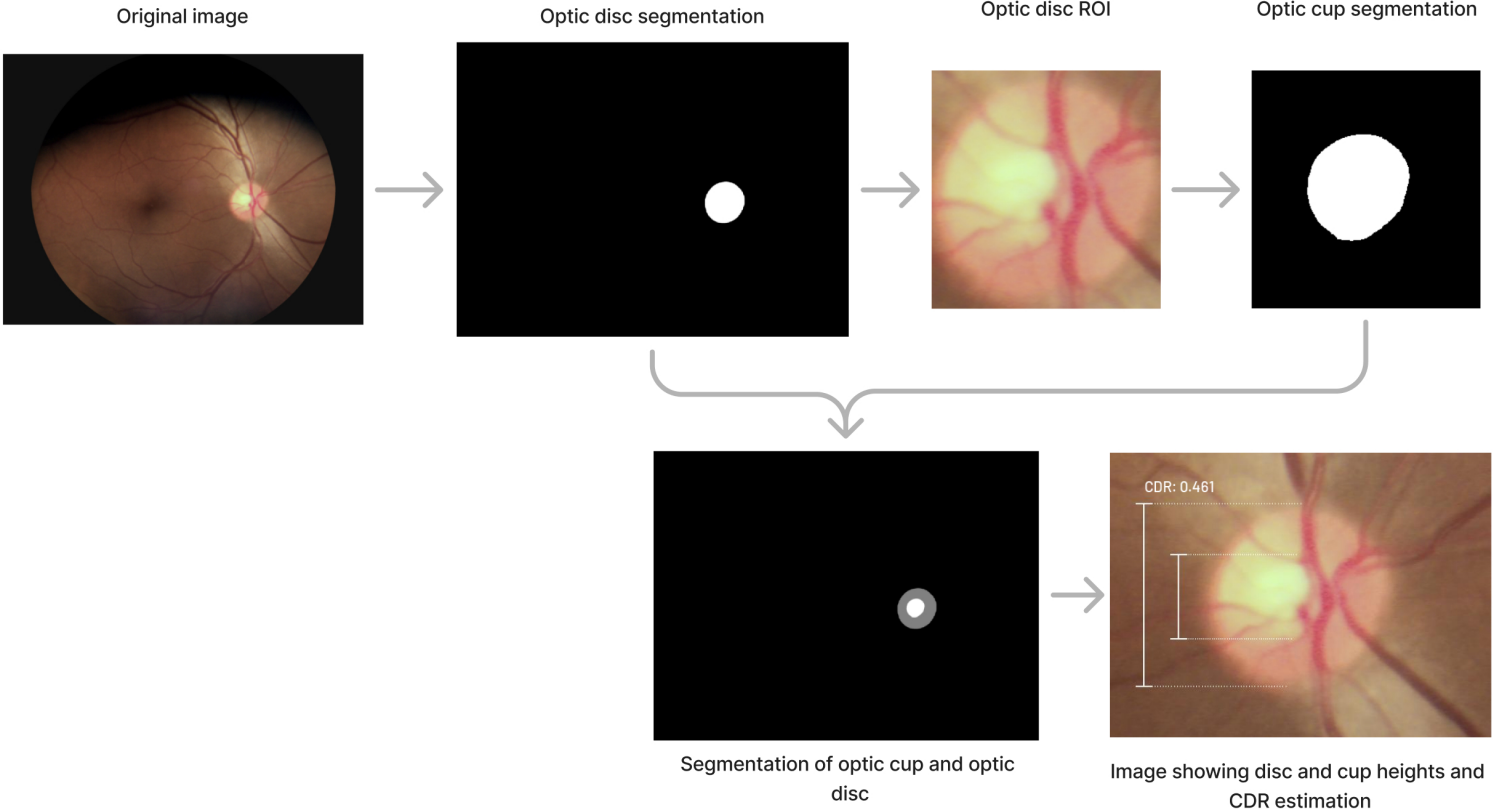
Process involved to determine CDR estimation.

A close-up of the optic disc is provided by the AI tool, with markings indicating the optic disc and optic cup heights, along with the CDR estimate. **Figure 4** graphically illustrates the process of estimating CDR. The final image corresponds to the explainability output provided by the AI tool.

The optic cup and optic disc segmentation models were trained on 18,446 images: 15,477 for training, 1,490 for validation, and 1,479 for internal testing. Corresponding masks were annotated by an ophthalmologist. Images were captured using various non-mydriatic fundus cameras, including the Horus 45° autofocus portable fundus camera from Jedmed, the Visuscout 100 from Zeiss, and the DRS from Centervue.

#### 2.4.3 Media opacities

Logical rules for detecting possible media opacities were defined with input from ophthalmologists and validated against patients with a diagnosis in an internal dataset. For media opacity assessment, the platform considers the following variables: image quality (resulting from the retinal analysis), patient age, and cataract-related symptoms, including blurry vision, changes in color perception, increased sensitivity to light, and difficulty seeing at night. As an example, if a patient is at least 60 years old, has blurry vision, and none of the images taken meet the quality threshold for retina evaluation, then the patient is classified as having possible media opacities.

### 2.5 Synergistic approach

The synergistic approach for both glaucoma suspects and retinal disease assessments considers the outcome as positive if either the residents or the AI tool identify a positive result.

## 3 Results

For both glaucoma suspects and the presence of retinal disease, we calculated accuracy, specificity, sensitivity, positive predictive value (PPV), and F1-score. To determine statistically significant differences between AI and residents, we calculated *p*-values using the bootstrap estimation method. For glaucoma suspect classification, we also computed the receiver operating characteristic (ROC) curve, using the maximum estimated CDR per patient as the model. Additionally, for CDR estimation, we evaluated absolute and relative errors, calculated the Pearson correlation coefficient (*r*), and presented Bland-Altman plots for residents and AI compared to ground truth values. Furthermore, we assessed media opacities and cataract classification by evaluating accuracy and Cohen’s kappa.

### 3.1 Glaucoma suspect and cup-to-disc ratio

The AI software analyzed CDR in 61.6% of patients, while in 38.4%, it determined that the image quality was insufficient for CDR analysis. In contrast, ground truth CDR values were obtained for 78.6% of patients. Ophthalmology residents, who conducted in-person evaluations, recorded CDR values for 95.4% of patients.

We compared the residents’ CDR annotations with their referrals for further glaucoma assessment. Among the referred patients, 89.4% had a CDR ≥ 0.6. Meanwhile, 73.6% of patients with a resident-assigned CDR ≥ 0.6 were referred to a glaucoma subspecialist or complementary testing.

The performance analysis was conducted on 245 patients (56.3%), specifically those for whom CDR values were available from the residents, the AI tool, and all three ophthalmologists. Metrics for glaucoma suspect classification are presented in **Table 1**. For AI classification, patients were identified as glaucoma suspects if their estimated CDR was 0.55 or higher, a threshold optimized for the maximum F1-score.

**Table 1:** Performance metrics for glaucoma suspect classification.

Classification Approach	Accuracy	Sensitivity	Specificity	PPV	F1 score
Resident diagnosis	82.9% (78.0%, 87.3%)	50.0% (35.3%, 64.8%)	90.5% (86.2%, 94.1%)	54.8% (39.1%, 69.7%)	52.3% (38.0%, 64.5%)
AI	**88.6%** (84.5%, 92.2%)	63.0% (50.0%, 77.3%)	**94.5%** (91.1%, 97.6%)	**72.5%** (57.1%,86.1%)	**67.4%** (55.2%, 77.8%)
Resident or AI	85.3% (80.4%, 89.4%)	**80.4%** (68.3%, 91.5%)	86.4% (81.2%, 90.7%)	57.8% (44.9%, 69.6%)	67.3% (55.4%, 76.0%)

The values considered for AI correspond to a high specificity point, where the best F1-score is met.

Bold values indicate the highest value for each evaluation metric.

When comparing the performance of AI to that of the ophthalmology residents, AI consistently outperformed the residents across all metrics. AI achieved 88.6% accuracy versus 82.9% for residents, 63.0% sensitivity versus 50.0% for residents, and 94.5% specificity versus 90.5% for residents. Statistically significant differences were found for accuracy (*p* = 0.016), PPV (*p* = 0.02), and F1-score (*p* = 0.026). However, differences in sensitivity (*p* = 0.116) and specificity (*p* = 0.062) were not statistically significant. The synergistic approach achieved the highest sensitivity (80.4%) and differed significantly from both AI alone (*p <* 0.001) and resident performance (*p <* 0.001).

**Figure 5:**
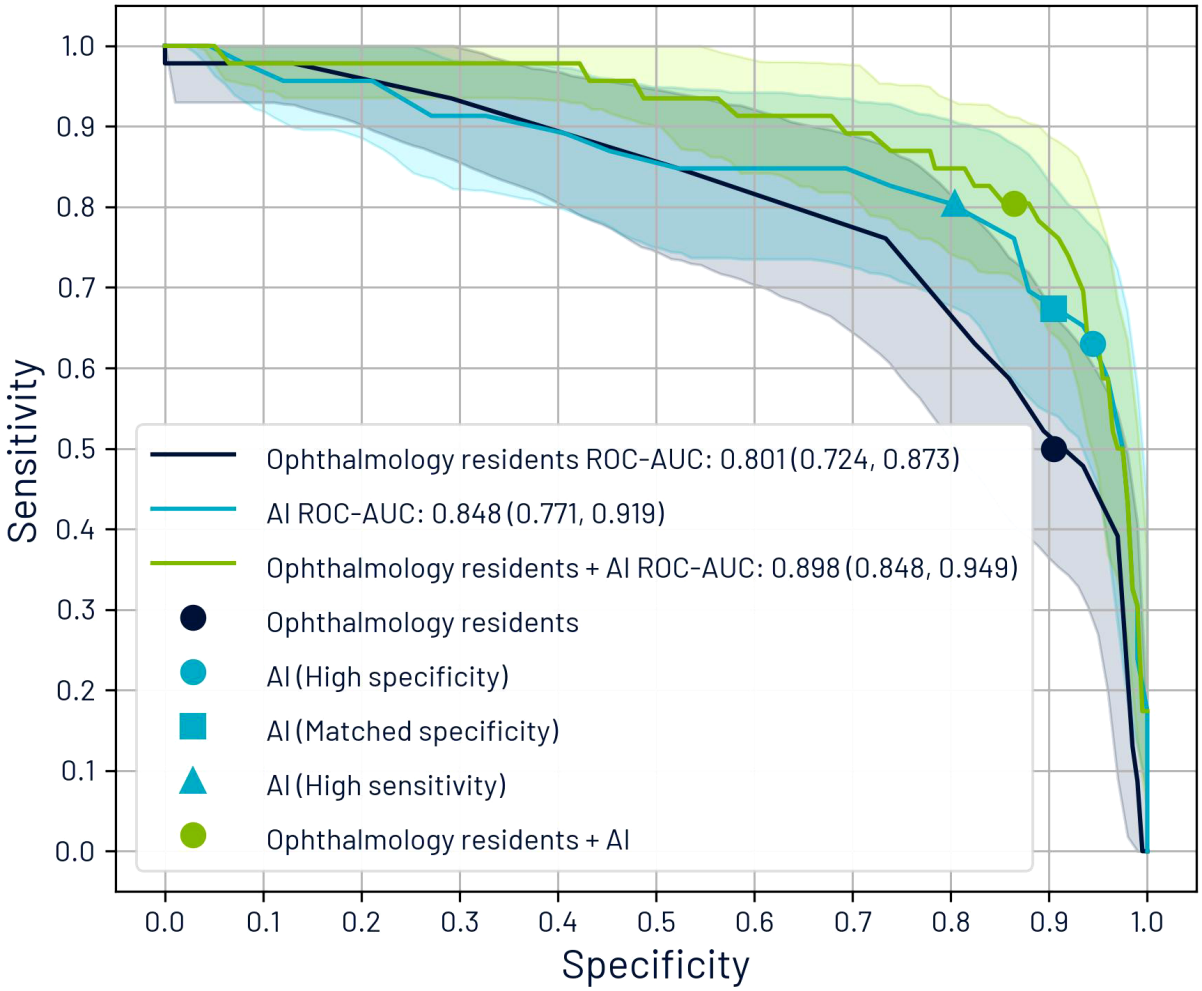
Receiver operating characteristic (ROC) curves comparing glaucoma suspect classification performance based on maximum cup-to-disc ratio (CDR) per patient. The graph displays individual performance curves for ophthalmology residents and AI systems, alongside a synergistic approach curve that integrates both assessment methods. The curve for the synergistic approach was derived by evaluating sensitivity and specificity metrics across multiple threshold combinations of resident and AI CDR estimations.

**Figure 5** presents the ROC curves for AI and residents, using the maximum patient-level CDR as the model for both. The ROC curve for the synergistic approach was generated by analyzing sensitivity and specificity across various combinations of cutoff points for residents and AI CDR estimates. The area under the ROC curve (ROC-AUC) was 0.848 for AI, 0.801 for residents, and 0.898 for the combined approach.

We identified five operating points: (1) the residents’ diagnoses, (2) AI’s high specificity point (optimal F1-score), (3) the point where AI’s specificity equaled that of the residents (67.4% sensitivity, 90.5% specificity), (4) the point where AI alone matched the sensitivity of the combined approach (80.4% sensitivity, 80.4% specificity), and (5) the synergistic approach results.

We further analyzed CDR estimates for the 362 eyes evaluated by all three ophthalmologists, the residents, and the AI tool. The mean absolute error for CDR estimation was 0.056 (SD: 0.042) for AI and 0.105 (SD: 0.074) for residents. The relative error was 10.9% (SD: 7.9%) for AI and 20.8% (SD: 14.4%) for residents. Performance differences were statistically significant (*p <* 0.001).

**Figure 6:**
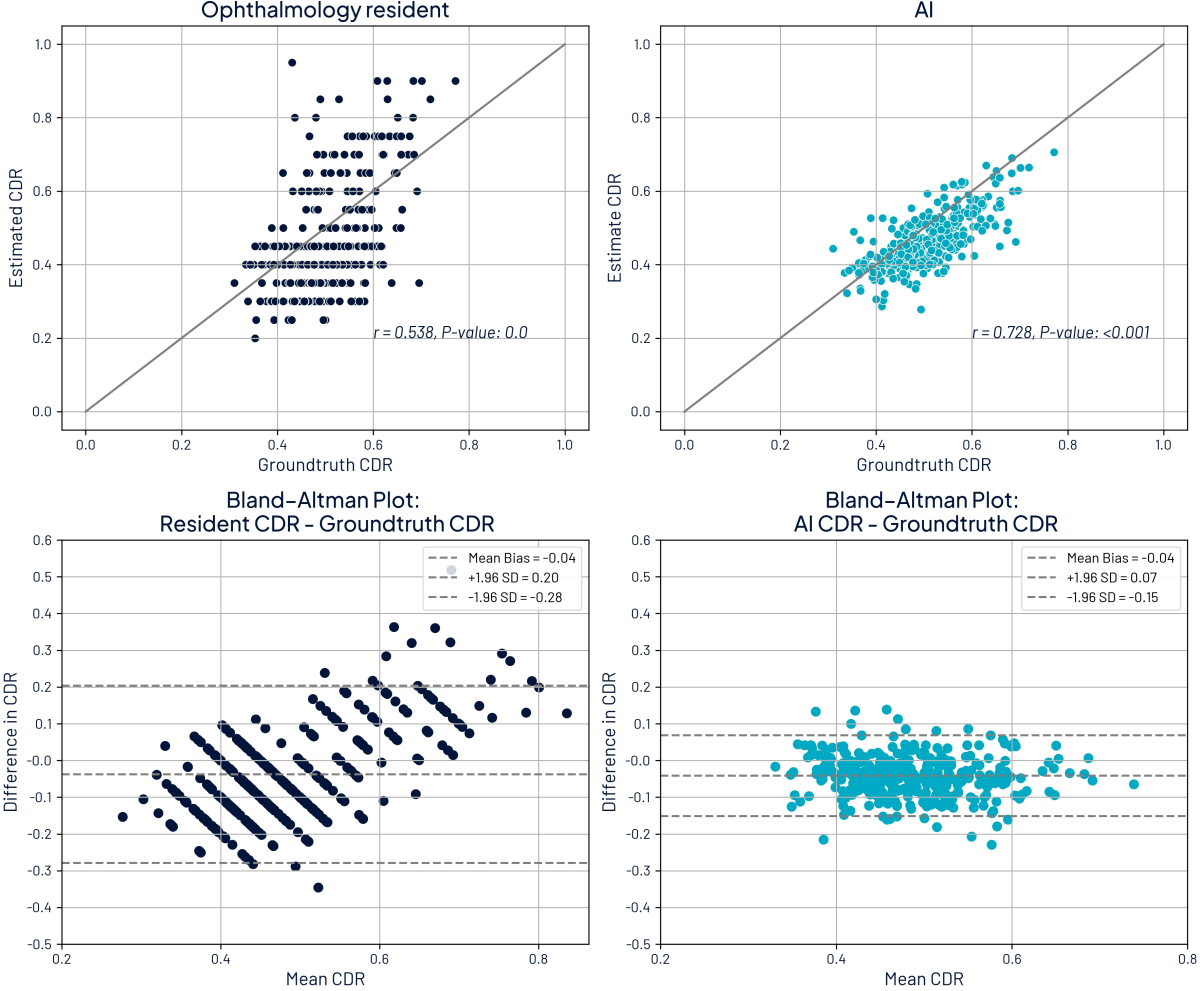
Comparison of cup-to-disc ratio (CDR) estimates with ground truth CDR and Bland-Altman plots. Top left: ground truth CDR against ophthalmology residents. Top right: ground truth CDR against AI estimates. Bottom left: Bland-Altman plot comparing residents to ground truth. Bottom right: Bland-Altman plot comparing residents to ground truth.

**Figure 6** illustrates the comparison between ground truth CDR values and estimates from both AI and residents, including both absolute estimates and Bland–Altman plots. A significant correlation was observed for both AI and residents (*p <* 0.001); however, AI achieved a higher Pearson correlation coefficient (*r* = 0.728) compared to the residents (*r* = 0.538). The Bland–Altman analysis reinforces this finding, with AI showing narrower limits of agreement (−0.15 to 0.07) than residents (−0.28 to 0.20), indicating greater consistency and lower variability in AI estimates. Moreover, the plot suggests a trend in residents’ estimations: underestimation of smaller CDRs and overestimation of larger ones, whereas AI maintains a relatively uniform variability across the range of values. Notably, both AI and residents exhibited a slight negative mean bias of −0.04, reflecting a mild underestimation relative to the ground truth.

While the AI tool provided more accurate CDR estimates and the synergistic approach outperformed residents alone, only 61.6% of patients could be evaluated with AI due to image quality limitations.

To better understand factors affecting image quality, we compared the prevalence of residents’ diagnoses between patients with ground truth CDR values (78.6%) and those without (21.4%). Diagnoses with at least a 25% relative increase in prevalence among patients without ground truth CDR included PM (6.7% vs. 0.5%), retinal detachment (10.0% vs. 1.2%), vitreous hemorrhage (7.5% vs. 1.5%), uveitis (0.8% vs. 0.2%), non-functional eye or prosthesis (5.8% vs. 1.5%), AMD (1.7% vs. 0.5%), DR (5.8% vs. 4.4%), and cataracts (9.2% vs. 7.2%). These findings suggest that retinal detachment, vitreous hemorrhage, and cataracts contribute to reduced image quality. Additionally, high myopia (associated with PM) may lead to blurry images, further impacting AI performance.

As an additional verification, we evaluated the impact of removing quality assessments on performance metrics. Specifically, we excluded the Inception V3 model, which is used to verify the quality of OD images. Without this step, 96.8% of patients would have a CDR assessment by AI. This resulted in having an AI assessment for all images with ground truth values. However, this would also include images deemed ungradable by ophthalmologists, making it impossible to evaluate performance against ground truth in these cases.

**Table 2** shows how performance metrics are affected when the quality component is missing. For those patients with sufficient quality images, sensitivity increases (67.4% vs. 63.0%), but specificity is lower (91.0% vs. 94.5%). In this subset, differences arise from additional images being evaluated by AI for a given patient instead of only evaluating those with sufficient quality. However, for the subset without gradable images, accuracy decreases to 76.7%, and there is a substantial increase in false positives, with specificity dropping to 80.3%.

**Table 2:** Performance metrics for AI in different subsets with groundtruth values without quality assessment component at a patient level.

Subset	Accuracy	Sensitivity	Specificity	PPV	F1 Score
All GT values	83.3% (79.5%, 87.1%)	66.2% (55.2%, 76.6%)	87.8% (83.9%, 91.6%)	58.8% (48.1%, 69.7%)	62.3% (52.5%, 70.7%)
Sufficient quality	**86.5%** (82.4%, 90.6%)	**67.4%** (53.1%, 80.0%)	**91.0%** (87.0%, 94.9%)	**63.3%** (7.5%, 28.3%)	**65.3%** (53.7%, 75.0%)
Insufficient quality	76.7% (67.8%, 84.4%)	66.7% (46.7%, 85.7%)	80.3% (70.4%, 89.2%)	55.2% 15.8%, 56.2%)	60.4% (42.3%, 74.1%)

Bold values indicate the highest value for each evaluation metric.

Moreover, the Pearson correlation between AI and resident assessments is 0.537 for images with sufficient quality. In contrast, for cases without ground truth, the correlation drops to 0.378, indicating greater discrepancies in assessments. This discrepancy may also correspond to larger errors in images that ophthalmologists considered ungradable.

### 3.2 Retinal disease

The analysis was performed on 395 patients (90.8%) where at least one eye was evaluated by AI to determine the presence of retinal disease. If the AI tool detected DR, ME, AMD, PM, or any other retinal disease, the case was considered positive for retinal disease.

**Table 3** presents performance metrics. We distinguished three categories for sensitivity analysis. The first category included the presence of all retinal disease, including those associated with mild stages of retinal disease (excluding tessellated fundus). The second category is associated with a medium or high risk of vision loss, and mild diseases are excluded. The third category corresponds to high risk of vision loss and encompasses findings such as vitreous hemorrhages, retinal detachment, and neovascularization. A complete list of what is included in each category is provided on **Supplementary Table S3** of **Supplementary Material**.

**Table 3:** Performance metrics for the presence of retinal disease.

Classification Approach	Accuracy	Sensitivity	Sensitivity medium risk and over	Sensitivity high risk	Specificity	PPV	F1 score
Resident diagnosis	75.2% (70.9%,79.7%)	51.9% (44.0%,59.9%)	63.0% (52.2%,73.3%)	80.5% (67.9%,92.1%)	90.4% (86.4%,93.9%)	77.9% (70.0%,85.4%)	62.3% (54.9%,69.0%)
AI	**88.1%** (84.8%, 91.1%)	76.3% (69.9%, 82.6%)	90.1% (83.5%, 96.1%)	**100.0%** (100.0%, 100.0%)	**95.8%** (93.1%, 98.3%)	**92.2%** (87.3%, 96.5%)	**83.5%** (78.7%,87.6%)
Resident or AI	86.1% (82.8%, 89.4%)	**84.0%** (78.4%, 89.9%)	**92.6%** (86.5%,97.7%)	**100.0%** (100.0%,100.0%)	87.4% (82.9%,91.2%)	81.4% (75.3%,86.7%)	82.6% (78.1%,86.9%)

Risk of vision loss is defined in terms of retinal lesions according to criteria on **Supplementary Table S3**.

Bold values indicate the highest value for each evaluation metric.

**Figure 7:**
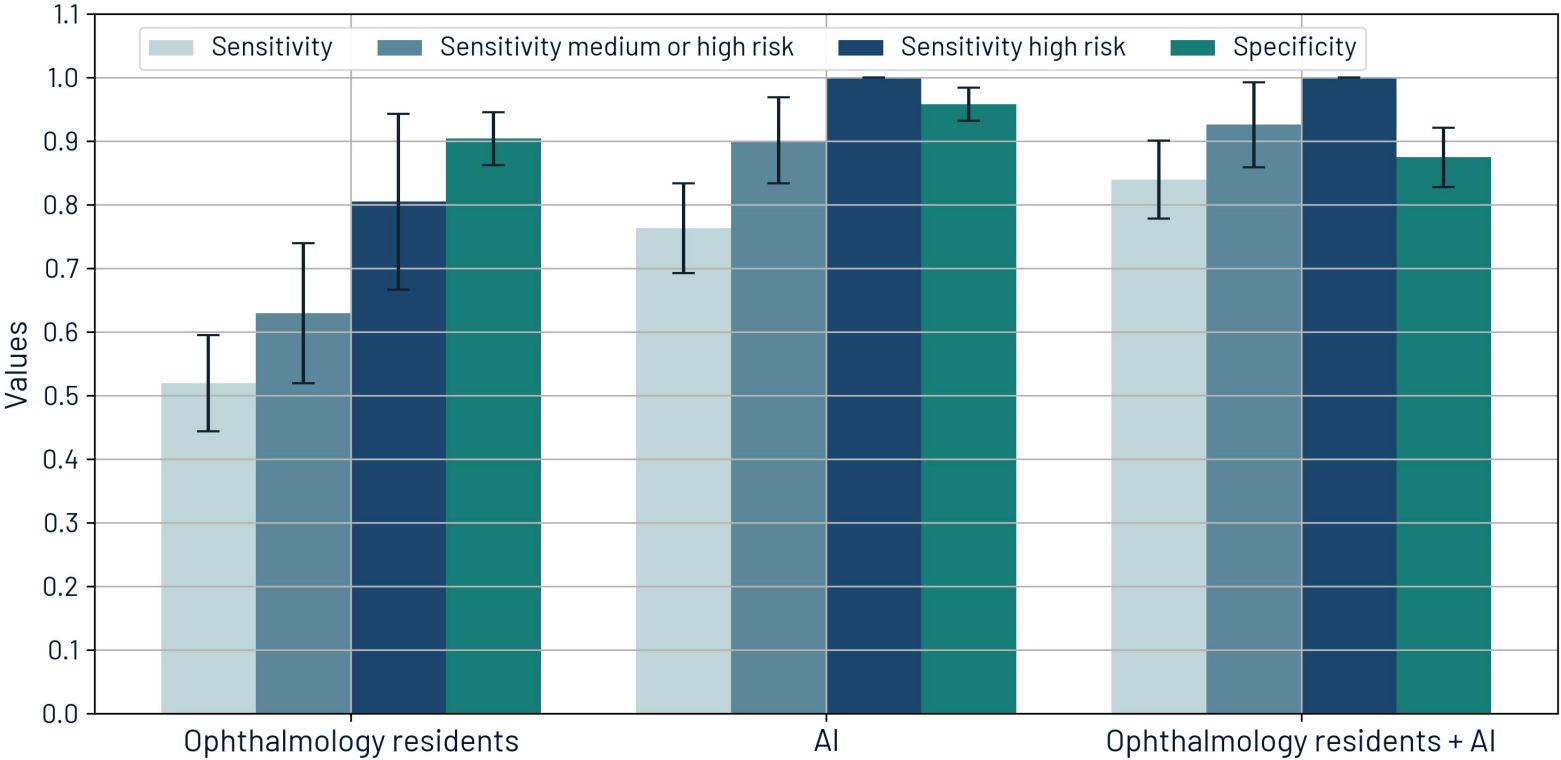
Sensitivities by risk of visual loss and specificity for retinal diseases for ophthalmology residents, AI, and the synergistic approach.

For both AI and ophthalmology residents, sensitivity improves as the risk of vision loss increases. Across all categories, AI demonstrated significantly higher sensitivity than the residents (*p <* 0.001). For all retinal diseases, AI achieved a sensitivity of 76.3%, significantly surpassing the resident’s sensitivity of 51.9%. For retinal disease associated with medium or high risk, AI’s sensitivity was 90.1% compared to residents’ 63.0%, and for high risk, AI reached 100% sensitivity versus 80.5%. Significant differences were observed across all metrics. Performance metrics are shown in **Figure 7**.

The synergistic approach resulted in improved sensitivity values: 84.0% for all retinal disease, 92.6% for medium or high risk, and 100% for high risk. PPV for this approach is 81.4%, which is better than the baseline PPV of the residents’ diagnoses.

Of the total patients analyzed for retinal disease, 9.2% had fundus images of insufficient quality, preventing AI-based analysis. Among these cases, remote ophthalmologists who reviewed the images classified 76.9% as ungradable. In contrast, ophthalmology residents conducting in-person evaluations categorized 25.6% of these patients as having cataracts and 15.4% as having either vitreous hemorrhage or retinal detachment—conditions that can obscure the retina and compromise image quality.

Additionally, Section 5 of the **Supplementary Material** presents an ablation study assessing the impact of image pre-processing and model outputs on ROC-AUC for retinal disease detection. It also includes a comparative table of ROC-AUC values for other technologies.

### 3.3 Cataract and media opacity comparison

During data collection, we gathered information on cataract diagnoses and referrals for cataract surgery. Additionally, media opacity detection is one of the possible outputs of the AI tool. Although there was no ground truth for cataract diagnosis, and the AI platform currently lacks the ability to differentiate cataracts from other media opacities, we compared the residents’ diagnoses with the AI tool’s results.

The percentage of agreement between ophthalmology residents and the AI tool was 86.0%. **Table 4** shows the confusion matrix that compares media opacity detection from AI and cataract diagnosis from ophthalmology residents.

**Table 4:** Confusion matrix for comparing media opacity detection from AI platform and cataract diagnosis from ophthalmology residents.

Resident diagnosis \ AI output	No media opacity (AI)	Media opacity (AI)
No cataract (resident)	360	35
Cataract (resident)	26	14

Despite a high percentage of agreement, the confusion matrix reveals poor agreement in terms of actual detection. This is reflected in the low Cohen’s Kappa value (*κ* = 0.237) (48). This low agreement may be due to the presence of opacities or cataract-related symptoms that correspond to different diseases. Also, the presence of some cataracts still allows fundus imaging with sufficient quality for analysis of the retina and therefore may not be considered by the AI tool as opacities.

## 4 Discussion

In this study, we evaluated the performance of an AI tool, the initial diagnoses made by ophthalmology residents, and a combined approach for retinal disease analysis, CDR estimation, and glaucoma suspect classification.

The AI tool demonstrated superior performance compared to ophthalmology residents in CDR estimation, achieving a lower average error (0.056 vs. 0.105, *p <* 0.001) and a higher Pearson correlation coefficient (0.728 vs. 0.538). This result was expected, as CDR estimation remains challenging even for experts (19, 23).

Glaucoma detection remains highly dependent on optic disc (OD) assessments, including a large CDR, significant differences in CDR between eyes, evaluation of the ISNT rule (disc rim thickness of inferior ≥ superior ≥ nasal ≥ temporal), and the presence of OD hemorrhages (30). CDR has been established as a key indicator of glaucoma (49). Our findings further support this, as 89.4% of patients referred for further glaucoma testing or to a glaucoma specialist had CDR estimates ≥0.6. In this study, we classified patients as glaucoma suspects if their CDR was ≥0.6 or if the CDR difference between both eyes exceeded 0.2, in accordance with established criteria for glaucomatous optic neuropathy assessment (30).

Under these criteria, AI achieved higher sensitivity and specificity (63.0% and 94.5%) compared to residents (50.0% and 90.5%). However, these differences were not statistically significant (*p* = 0.116 and *p* = 0.062).

When employing a synergistic approach, sensitivity significantly increased to 80.4% (*p <* 0.001) while maintaining a specificity of 86.4%, highlighting the potential benefit of integrating AI with resident assessments in clinical practice, reducing false negatives with a minor effect on false positives. Furthermore, AI alone could serve as a valuable tool in primary care settings where patients at risk of developing ophthalmic conditions are evaluated by general physicians or are not evaluated at all.

Based on these findings, implementing this AI tool could standardize CDR measurements and enhance glaucoma suspect detection. However, we did not assess whether the AI tool’s explainability features could assist residents in determining CDR measurements. Future research should explore the potential benefits of incorporating visual aids for CDR estimation.

Other imaging techniques, such as optical coherence tomography (OCT), provide additional biomarkers that correlate more strongly with glaucoma than CDR, such as retinal nerve fiber layer thickness (50). However, OCT evaluations are typically conducted after an initial screening, whereas this study focuses on fundus imaging and first-time assessments performed by residents.

For retinal disease detection, there was a notable difference in sensitivity between AI and residents. For retinal diseases with a medium or high risk of visual loss, AI achieved a sensitivity of 90.1%, compared to 63.0% for residents. Even when sensitivity increased for high-risk findings, residents only reached 80.5% sensitivity compared to the 100% achieved by AI. AI also demonstrated higher specificity, resulting in an overall superior performance.

Furthermore, AI demonstrated statistically significant superiority across all metrics. Although not all patients require referral for further testing or to specialist evaluation, high sensitivity is crucial for optimizing referral decisions and providing more effective recommendations regarding self-care and follow-up.

It is also important to acknowledge AI’s limitations, particularly in terms of false positives and false negatives. For glaucoma detection, AI achieved a specificity of 94.5%, indicating a low rate of false positives. However, its sensitivity was 63.0%, reflecting a relatively high number of false negatives. Nonetheless, this remains an improvement over residents, whose sensitivity was 50.0%, aligning with current standards for first-time assessments in tertiary care. While further training could enhance AI’s sensitivity, its performance already meets or exceeds the current standard of care.

For retinal disease detection, AI achieved a specificity of 95.8% and a PPV of 92.2%, indicating that only 7.8% of identified cases were false positives. AI’s sensitivity for retinal diseases with medium to high risk of vision loss was 90.1%, meaning that only 9.9% of such cases were missed. In comparison, residents failed to detect 37.0% of these cases. Thus, AI not only improves sensitivity but also reduces both false positives and false negatives compared to resident assessments. This improvement was particularly pronounced when examining high-risk cases.

For these high-risk cases, AI’s sensitivity reached 100%, while residents’ sensitivity was only 80.5%. Although the confidence interval for AI was 100% to 100% using the bootstrap resampling method, it does not guarantee perfect generalization. Instead, it reflects no observed variability in sensitivity within this specific sample. This underscores the importance of validating AI models on larger datasets.

The implementation of the AI tool holds significant promise for enhancing sensitivity in retinal disease assessments and improving the accuracy of CDR measurements. However, it is crucial to emphasize that the final diagnosis must always be based on a comprehensive ophthalmological evaluation.

Poor image quality significantly affects AI’s ability to assess the OD and the retina. For OD evaluation, AI successfully estimated the CDR in only 61.6% of cases, whereas all three ophthalmologists deemed 78.6% of images of sufficient quality. This suggests that adjusting AI’s quality criteria could improve OD evaluations. Alternatively, using higher resolution fundus cameras may enhance image quality, though this could increase implementation costs. However, even with improved imaging, quality issues may persist in patients with media opacities or high myopia, as physiological variations can make obtaining high-quality images challenging.

Although removing quality checks increases the number of patients receiving an AI evaluation for CDR and glaucoma, it also increases the number of false positives, reflected in a lower specificity. This trade-off underscores the importance of incorporating quality controls to ensure diagnostic reliability. Moreover, performance cannot be meaningfully assessed on images without ground truth, as these were deemed ungradable by experts.

Beyond OD evaluation, image quality also affects retinal disease detection. In cases where AI deemed images ungradable, 76.9% were also classified as ungradable by remote ophthalmologists, reinforcing the inherent limitations of remote assessments when image quality is poor. Furthermore, 25.6% of these patients had cataracts and 15.4% had vitreous hemorrhage or retinal detachment, conditions that obscure the retina and compromise image quality. Given the significant role of media opacities in image degradation, in-person ophthalmologic evaluations remain essential when AI determines that an image is of insufficient quality.

This study focused on fundus images for both AI analysis and ground truth values. However, OCT may offer better visualization of retinal layers, enhancing lesion assessment and annotation accuracy. This could provide a more comprehensive evaluation of both the AI system and ophthalmology residents. Although OCT was not used—due to hospital protocols limiting its use to selected cases—future studies could benefit from comparing OCT-based ground truth to further validate and refine AI performance.

Ophthalmologists conduct thorough evaluations that extend beyond digital imaging, encompassing the peripheral retina, anterior segment structures, and media opacities that may obscure image clarity. These critical aspects of clinical evaluation cannot be fully replicated by AI. Additionally, the final decision-making process—whether to refer a patient to a subspecialist, request further studies, or determine that no referral is needed—remains a fundamental responsibility of the clinician.

Despite these limitations, AI serves as a valuable tool in retinal disease assessment by standardizing CDR estimates, improving sensitivity, and guiding decisions regarding additional examinations, referrals, and follow-ups.

Currently, Mexico and other Latin American countries are in the process of developing regional regulations and best practice guidelines for AI-based tools in healthcare, particularly in the context of Software as a Medical Device (SaMD). Our study contributes to this ongoing discussion by highlighting the potential of AI in healthcare systems, especially in regions with limited resources for early disease detection and vision loss prevention.

While this study successfully evaluated the comparative performance of AI-assisted ophthalmic assessments and its potential to enhance diagnostic sensitivity, several key directions for future research remain.

The study design prioritized establishing baseline performance metrics before introducing AI into direct patient care. Future research should focus on integrating AI into clinical workflows, assessing its impact on patient outcomes, and exploring its role in ophthalmology resident training. Specifically, future studies should (1) conduct a dedicated user study to examine how ophthalmology residents benefit from AI assistance in clinical settings and evaluate the clinical interpretability of explainability features such as heatmaps generated using GradCam and SmoothGrad, (2) document real-world case studies in which AI directly influences diagnosis or treatment decisions, (3) expand the dataset to include a more diverse patient population, and (4) conduct longitudinal studies to assess the real-world clinical performance of the system over extended periods.

These next steps will provide critical insights as we transition from performance assessment to the responsible clinical integration of AI.

## Data Availability

The raw data supporting the conclusions of this article will be made available by the authors, without undue reservation.
