# Supplementary material for: Synergistic AI-resident approach achieves superior diagnostic accuracy in tertiary ophthalmic care for glaucoma and retinal disease
Source: Front Ophthalmol (Lausanne). 2025 May 19;5:1581212. doi: 10.3389/fopht.2025.1581212 (PMC12127187; doi:10.3389/fopht.2025.1581212)
Supplement: Supplementary file 1 [file DataSheet1.pdf]

Supplementary Material

1 VARIABLES COLLECTED DURING THE SCREENING PROCESS

The list of variables collected at Conde de Valenciana Centro during the screening porcess are shown on table 1.

Code	Definition	Type	Values
expediente	Medical record ID.	text	-
patient_age	Age.	numeric	0-120
postalCode	Zip Code	text	-
gender	Sex assigned at birth.	categorical	1: Male<br>2: Female
diabetes	Diagnosed diabetes.	boolean	0: No<br>1: Yes
diabetesYears	Time with diagnosed diabetes.	numeric	0-25
hypertension	Diagnosed hypertension	boolean	0: No<br>1: Yes
hypertensionYears	Time with diagnosed hypertension.	numeric	0-25
lastVisitOphthamologist	Date of last ophthalmic evaluation	categorical	1: Within the last 12 months<br>2: More than a year ago<br>3: Never
sight_loss_in_family	Family history of vision loss	boolean	0: No<br>1: Yes
visionAffectingQualityLife	Patient perceives that visual conditions interfere with daily activities	boolean	0: No<br>1: Yes
SY-OPH-CAT-1	Blurry or foggy vision even with glasses	boolean	0: No<br>1: Yes
SY-OPH-CAT-2	Changes in color perception	boolean	0: No<br>1: Yes
SY-OPH-CAT-3	Increased sensitivity to light or halos	boolean	0: No<br>1: Yes
SY-OPH-CAT-4	Difficulty to see at night	boolean	0: No<br>1: Yes
SY-OPH-OT-2	Perceived flashes or light streaks	boolean	0: No<br>1: Yes
SY-OPH-OT-3	Eye pain	boolean	0: No<br>1: Yes
SY-OPH-OT-4	Spots or patches that persist within vision all the time	boolean	0: No<br>1: Yes
SY-OPT-PR-1	Difficulty seeing up close or reading small print	boolean	0: No<br>1: Yes
symptomDevelop	How did the changes in the patient's vision appeared.	categorical	1: Suddenly<br>2: Progressively

Table S1. Variables collected in screening process.

2 PATIENT FLOW DIAGRAM

The diagram in Figure S1 shows which patients were included and which were excluded for retinal disease analysis and glaucoma analysis.

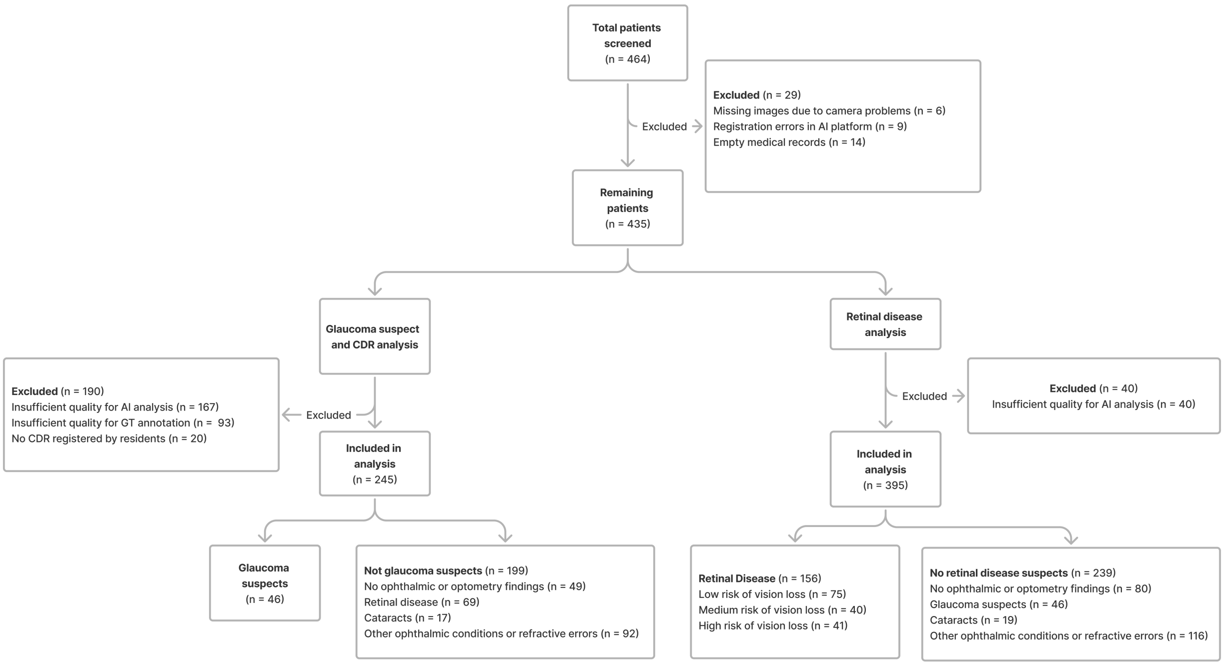

Figure S1. Patient flow, indicating which patients were excluded and which were included for each analysis.

3 RETINAL FINDINGS ANNOTATED IN FUNDUS IMAGES

Retinal findings that were annotated by ophthalmologists in training and in evalutaion process are shown in Table S2.

Category	Findings
Atrophies	S-AT-1: Diffuse chorioretinal atrophy<br>S-AT-2: Patchy chorioretinal atrophy<br>S-AT-3a: Geographic or macular atrophy involving the fovea<br>S-AT-3b: Geographic or macular atrophy not involving the fovea
Drusen	S-DR-1a: Less than 20 small drusen<br>S-DR-1b: 20 or more small drusen<br>S-DR-2a: Less than 20 medium drusen<br>S-DR-2b: 20 or more medium drusen<br>S-DR-3: Large drusen
Exudates	S-EX-1a: Hard exudates within one disk diameter of macula center.<br>S-EX-1b: Hard exudates further than one disk diameter from the macula center, but within the temporal arcades<br>S-EX-1c: Hard exudates outside of the temporal arcades<br>S- EX-2: Soft exudates
Hemorrhages and Microaneurysms	S-HM-1: Microaneurysms<br>S-HM-2: Vitreous hemorrhage<br>S-HM-3: Preretinal hemorrhage<br>S-HM-4: Superficial hemorrhage<br>S-HM-5a: Less than 20 intra-retinal hemorrhages<br>S-HM-5b: 20 or more intra-retinal hemorrhages<br>S-HM-6: Subretinal hemorrhage
Vascular anomalies	S-VA-1: Intraretinal microvascular abnormalities<br>S-VA-2: Venous beading<br>S-VA-3: Neovascularization<br>S-VA-4: Changes in the arteriolar light reflex<br>S-VA-5: Arteriovenous crossings<br>S-VA-6: Focal narrowing of retinal arterioles<br>S-VA-7: Generalised narrowing of retinal arterioles<br>S-VA-X: Other microvascular abnormalities
Other retinal findings	S-OT-1: Retinal pigmentary epithelium changes<br>S-OT-2: Choroidal neovascular membrane<br>S-OT-4: Lacquer cracks<br>S-OT-5: Fuch's spot<br>S-OT-6: Disciform scar<br>S-OT-7: Epiretinal membrane<br>S-OT-8: Macular hole<br>S-OT-9: Rhegmatogenous retinal detachment<br>S-OT-10: Tractional retinal detachment<br>S-OT-11: Central or branch artery occlusion<br>S-OT-12: Central or branch vein occlusion<br>S-OT-XR: Other pathological signs in retina
Non pathological findings	S-NP-1: Tessellated fundus<br>S-NP-2: Retinal fiber layer myelination<br>S-NP-3: Choroidal nevus<br>S-NP-4: Panretinal photocoagulation traces<br>S-NP-X: Other non-pathological findings
Insufficient quality	S-Q-1: Insufficient quality to assess the retina

Table S2. Findings annotated during the labeling process.

4 LOGICAL RULES TO DETERMINE PREDIAGNOSIS AND RISK OF VISUAL LOSS FROM FINDINGS IN FUNDUS IMAGES

To define DR stages we used the International Clinical Diabetic Retinopathy and Diabetic Macular Edema Disease Severity Scales (ICDR) (Wilkinson et al., 2003).

According to the Guidelines on Diabetic Eye Care, DME is defined as retina thickening, however this cannot be measured on a 2D fundus images (Wong et al., 2018). Thus, we use hard exudates as a proxy of DME, since they are a sign of current or previous DME (Wong et al., 2018). Moreover, Litvin *et al* found that detecting hard exudates within one disc diameter (DD) from the fovea had a 93.8% sensitivity for clinically significant DME (Litvin et al., 2014). Therefore, we consider hard exudates within one DD as a proxy for center-involving diabetic macular edema, and other hard exudates within the macula area as a proxy for non-center-involving diabetic macular edema.

For AMD there are several definitions and severity scales, such as (Bird et al., 1995; Age-Related Eye Disease Study Research Group, 1999; Davis et al., 2005; Ferris et al., 2005; Seddon et al., 2006; Coleman et al., 2008; Klein et al., 2014; Flaxel et al., 2020). Most of them consider drusen, retinal pigmentary epithelium (RPE) changes, geographic atrophy; and choroidal neovascularization as well as other signs of neovascular maculopathy such as RPE detachment. We considered a simplified version of the Preferred Practice Pattern (PPP) scale used by Flaxel *et al* (Flaxel et al., 2020). We considered the mild stage to have more than 20 small drusen, from 1 to 19 medium drusen, or RPE changes. The moderate stage included more than 20 medium drusen, at least one large druse, or non-central geographic atrophy. For the advanced stage we considered either geographic atrophy involving the foveal center or a choroidal neovascular membrane additional to drusen or RPE changes.

To classify pathological myopia we used the categorization by Ohno-Matsui *et al* considering the 4 stages and an additional stage if any of the “plus” lesions was present.(Ohno-Matsui et al., 2015)

To determine which retinal findings corresponded to the different levels of risk of visual loss, we consulted two retina specialists. Findings on the medium or high risk category, correspond to those for which both retina experts agreed a consultation with a retina expert was required. Findings on the high risk of visual loss, were those that could require treatment. The latter, considering hard exudates on the macular area as a proxy for macular edema, and excluding vitamin prescription for AMD within the treatments.

The criteria for risk classification were informed by follow-up and treatment guidelines, including those from the Mexican Institute of Social Security (IMSS, 2014) and the Age-Related Macular Degeneration (AMD) Preferred Practice Pattern (Flaxel et al., 2020). For risk classification, findings that do not require evaluation by a retina specialist and could be followed up annually or at longer intervals were classified as low risk. Findings classified as high risk were those that potentially required treatment, such as anti-VEGF injections or surgery (excluding vitamin prescription for AMD), and included cases with hard exudates in the macular area, considered a proxy for macular edema (Litvin et al., 2014). Medium risk findings were those that require follow-up within one year according to clinical guidelines but do not require immediate treatment. These criteria were reviewed and validated by two retina specialists.

The corresponding rules that determine possible prediagnosis and risk of visual loss are presented on Table S3.

Disease	Severity	Rules
Diabetic Retinopathy	Mild	S-HM-1
	Moderate	(S-HM-1 **AND** (S-EX-1a **OR** S-EX-1b **OR** S-EX-1c **OR** S-EX-2)) **OR** S-HM-5a
	Severe	((S-HM-1 **OR** S-HM-5a) **AND** (S-VA-1 **OR** S-VA-2)) **OR** S-HM-5b
	Proliferative	S-VA-3 **OR** S-HM-2 **OR** S-HM-3
Macular Edema	Non-central	S-EX-1b
	Central	S-EX-1a
Age Macular Degeneration	Mild	S-DR-1b **OR** S-DR-2a **OR** S-OT-1
	Intermediate	S-DR-2b **OR** S-DR-3 **OR** (S-AT-3b **AND** (S-DR-1 **OR** S-DR-2a **OR** S-OT-1))
	Advanced	(S-AT-3a **OR** S-OT-2) **AND** (S-DR-1 **OR** S-DR-2a **OR** S-DR-2b **OR** S-DR-3 **OR** S-OT-1)
Pathological Myopia	1	S-NP-1
	2	S-NP-1 **AND** S-AT-1
	3	S-NP-1 **AND** S-AT-2
	4	S-NP-1 **AND** (S-AT-3a **OR** S-AT-3b)
	PLUS	S-NP-1 **AND** (S-OT-2 **OR** S-OT-4 **OR** S-OT-5)
Retinal findings	-	S-VA-3<br>**OR** S-AT-1 **OR** S-AT-2 **OR** S-AT-3a **OR** S-AT-3b<br>**OR** S-DR-1b **OR** S-DR-2a **OR** S-DR-2b **OR** S-DR3<br>**OR** S-EX-1a **OR** S-EX-1b **OR** S-EX-1c **OR** S-EX-2<br>**OR** S-HM-1 **OR** S-HM-2 **OR** S-HM-3 **OR** S-HM-4 **OR** S-HM-5a **OR** S-HM-5b **OR** S-HM-6<br>**OR** S-OT-1 **OR** S-OT-2 **OR** S-OT-4 **OR** S-OT-5 **OR** S-OT-6 **OR** S-OT-7 **OR** S-OT-8 **OR** S-OT-9 **OR**<br>S-OT-10 **OR** S-OT-11 **OR** S-OT-12 **OR** S-NP-4 **OR** S-OT-XR
Medium or high risk of visual loss	-	S-VA-3<br>**OR** S-AT-2 **OR** S-AT-3a **OR** S-AT-3b<br>**OR** S-DR-2b **OR** S-DR3<br>**OR** S-EX-1a **OR** S-EX-1b<br>**OR** S-HM-2 **OR** S-HM-3 **OR** S-HM-5a **OR** S-HM-5b **OR** S-HM-6<br>**OR** S-OT-1 **OR** S-OT-2 **OR** S-OT-6 **OR** S-OT-8 **OR** S-OT-9 **OR**<br>S-OT-10 **OR** S-OT-12 **OR** S-NP-4
High risk of visual loss	-	S-VA-3<br>**OR** S-EX-1a **OR** S-EX-1b<br>**OR** S-HM-2 **OR** S-HM-3 **OR** S-HM-6<br>**OR** S-OT-2 **OR** S-OT-8 **OR** S-OT-9 **OR**<br>S-OT-10 **OR** S-OT-12

Table S3. Severity grades for DR, ME, AMD, and PM and the lesions in retina that conform them. For lesions in retina we exclude vascular anomalies other than neovascularization (S-VA-3). We also excluded non-pathological findings, except for panretinal photocoagulation traces, which may require continuous assessment by a retina specialist.

5 ABLATION STUDY FOR RETINAL DISEASE MODEL

The AI platform for retinal disease detection consists of multiple outputs, each classifying the presence of different ophthalmic conditions, as well as an overall detection of retinal lesions. In previous analyses, a positive prediction for any disease was considered an indicator of retinal disease presence. However, not all outputs may contribute equally to general detection. Therefore, we assess how the inclusion or exclusion of specific outputs impacts the overall model performance in detecting retinal disease.

Additionally, the methodology requires a specialized preprocessing step before applying the model. To evaluate the significance of this preprocessing, we analyze the model's performance when using only basic image resizing, comparing it to the standard preprocessed approach. This helps quantify the contribution of preprocessing to the model's accuracy and robustness.

ROC curves and corresponding AUC values were calculated for each scenario and are presented in Figure S2. The results highlight the crucial role of preprocessing in model performance. With preprocessing, the ROC-AUC was 0.938, whereas without preprocessing, it dropped significantly to 0.647. This demonstrates that proper preprocessing enhances the model's ability to detect retinal disease.

When evaluating individual outputs separately, the retinal lesions classifier achieved the highest ROC-AUC (0.937), followed by diabetic macular edema (0.907) and age-related macular degeneration (0.85). This suggests that while each output contributes to the overall disease detection, some outputs carry more predictive power than others.

For detecting the general presence of retinal lesions, the model could rely primarily on the retinal lesions output, as this output was trained to detect a broader range of retinal abnormalities compared to disease-specific outputs. When considering pairs of disease-specific outputs, the best-performing pair was DR and AMD (ROC-AUC: 0.903), followed by ME and AMD (ROC-AUC: 0.894). This could be explained by the distinct types of lesions present in DR and AMD, or in ME and AMD. In contrast, the pair with the lowest ROC-AUC was DR and ME (0.861), likely because ME and DR frequently co-occur, meaning that combining them does not contribute significantly to detecting additional lesions.

The ablation study is focused mainly on the performance of detecting retinal diseases in general. However, disease-specific are optimized for specific pathologies. Consequently, their ROC-AUC values are higher when evaluated for their respective diseases compared to their performance in overall disease detection as can be seen in Figure S3.

The ROC-AUC values obtained for retinIA in this study are comparable to those previously reported for other AI technologies based on fundus images. For diabetic retinopathy (DR), ROC-AUC values for fundus image-based models range from 0.915 to 0.959 (Bhaskaranand et al., 2019; Nissen et al., 2023; Arenas-Cavalli et al., 2022; Ting et al., 2017), with retinIA achieving an ROC-AUC of 0.935.

For glaucoma and AMD, retinIA shows lower ROC-AUC values compared to EyeArt, Retinalyze, and SELENA+ (Ramachandra et al., 2015; Mendez-Hernandez et al., 2023; Ting et al., 2017). However, it is important to note that these evaluations were conducted on different datasets from different populations, which may affect direct comparability between studies.

Metrics for macular edema (ME), pathological myopia (PM), and general retinal lesions (RL) are only available for retinIA. Additionally, PM and General RL are exclusive outputs of retinIA, highlighting its broader diagnostic capabilities.

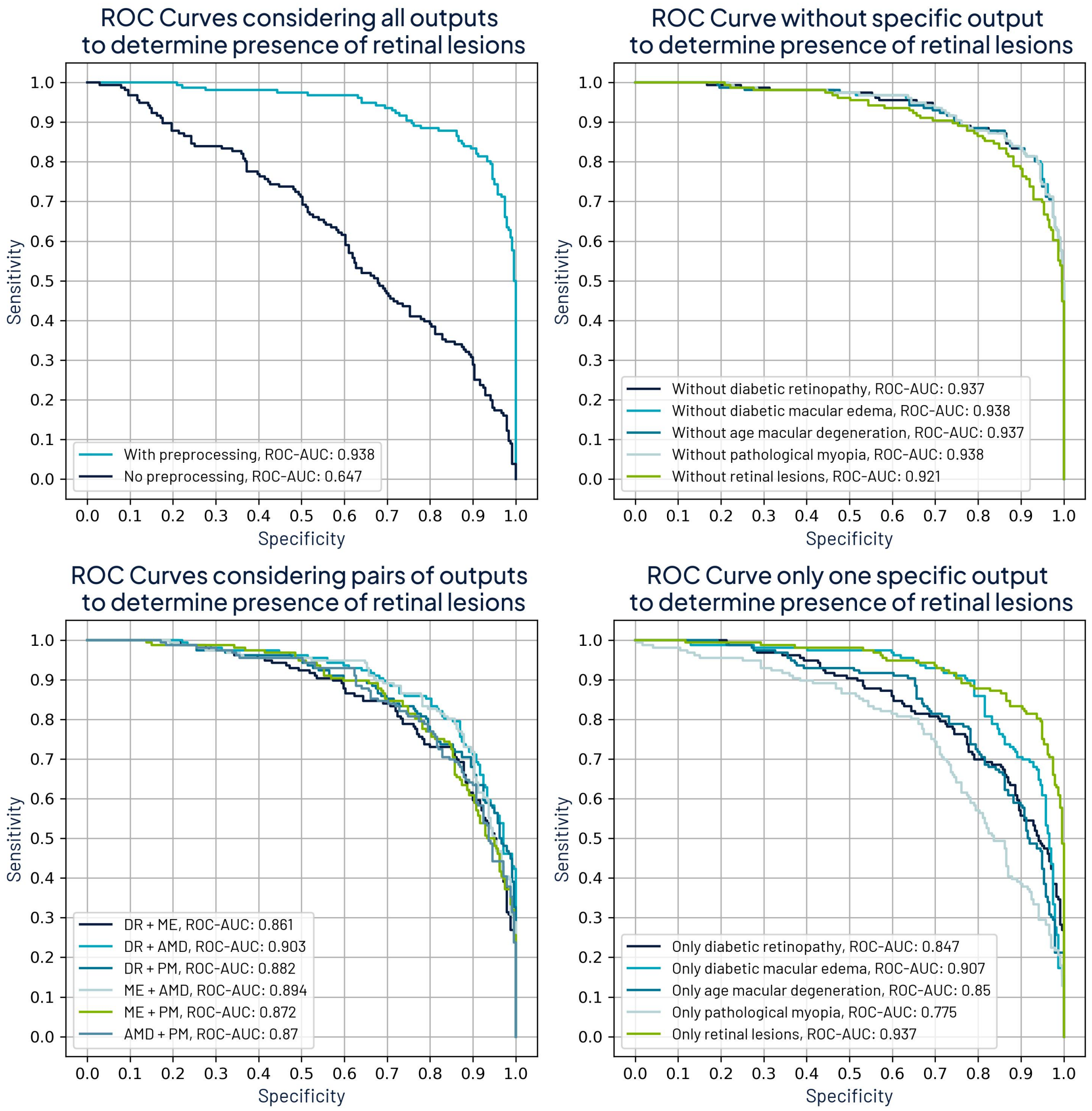

Figure S2. ROC Curves for the Ablation Study. (Top-Left) Comparison of models with and without preprocessing. (Top-Right) Performance impact when removing specific disease outputs. (Bottom-Left) ROC curves for models trained with disease pairings. (Bottom-Right) ROC curves when using only one disease-specific output for prediction.

Other deep learning systems for ophthalmology, such as Google DeepMind for AMD and AMD progression, have been evaluated. However, this system is based on OCT scans, making it not directly comparable to technologies that rely on fundus images (Yim et al., 2020).

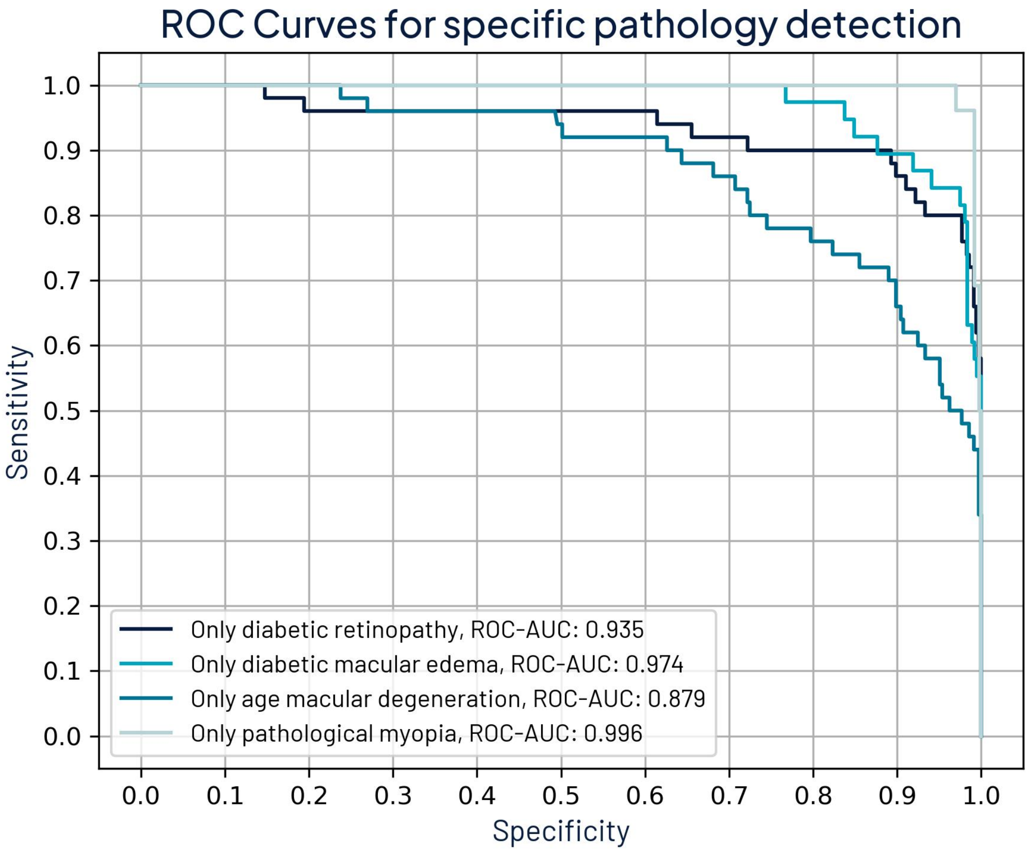

Figure S3. Performance of each output in detecting its corresponding pathology, showing improved AUC values when evaluated for their specific disease.

	DR	ME	AMD	PM	General RL	Glaucoma
**retinIA**	0.935	0.974	0.879	0.996	0.937	0.848
**EyeArt**	0.959	-	0.900	-	-	Not reported
**Retinalyze**	0.934	-	Not reported	-	-	0.953
**DART**	0.915	-	-	-	-	
**SELENA+**	0.936	Not reported	0.931	-	-	0.942

Table S4. Comparison of AI model performance for different retinal diseases (Bhaskaranand et al., 2019; Ramachandra et al., 2015; Nissen et al., 2023; Mendez-Hernandez et al., 2023; Arenas-Cavalli et al., 2022; Ting et al., 2017).

REFERENCES

Wilkinson CP, Ferris FL, Klein RE, Lee PP, Agardh CD, Davis M, et al. Proposed international clinical diabetic retinopathy and diabetic macular edema disease severity scales. *Ophthalmology* **110** (2003) 1677–82. doi:10.1016/S0161-6420(03)00475-5.

Wong TY, Sun J, Kawasaki R, Ruamviboonsuk P, Gupta N, Lansingh VC, et al. Guidelines on Diabetic Eye Care: The International Council of Ophthalmology Recommendations for Screening, Follow-up, Referral, and Treatment Based on Resource Settings. *Ophthalmology* **125** (2018) 1608–1622. doi:10.1016/j.ophtha.2018.04.007.

Litvin TV, Ozawa GY, Bresnick GH, Cuadros JA, Muller MS, Elsner AE, et al. Utility of hard exudates for the screening of macular edema. *Optometry and Vision Science* **91** (2014) 370–375. doi:10.1097/OPX.

0000000000000205.

Bird AC, Bressler NM, Bressler SB, Chisholm IH, Coscas G, Davis MD, et al. An international classification and grading system for age-related maculopathy and age-related macular degeneration. The International ARM Epidemiological Study Group. *Survey of Ophthalmology* **39** (1995) 367–374. doi:10.1016/s0039-6257(05)80092-x.

Age-Related Eye Disease Study Research Group. The Age-Related Eye Disease Study (AREDS): design implications. AREDS report no. 1. *Controlled Clinical Trial* **20** (1999) 573–600. doi:10.1016/s0197-2456(99)00031-8.

Davis MD, Gangnon RE, Lee LY, Hubbard LD, Klein BE, Klein R, et al. The Age-Related Eye Disease Study severity scale for age-related macular degeneration: AREDS Report No. 17. *Archives of ophthalmology (Chicago, Ill.: 1960)* **123** (2005) 1484–1498. doi:10.1001/archopht.123.11.1484.

Ferris FL, Davis MD, Clemons TE, Lee LY, Chew EY, Lindblad AS, et al. A simplified severity scale for age-related macular degeneration: AREDS Report No. 18. *Archives of ophthalmology (Chicago, Ill.: 1960)* **123** (2005) 1570–1574. doi:10.1001/archopht.123.11.1570.

Seddon JM, Sharma S, Adelman RA. Evaluation of the clinical age-related maculopathy staging system. *Ophthalmology* **113** (2006) 260–266. doi:10.1016/j.ophtha.2005.11.001.

Coleman HR, Chan CC, Ferris FL, Chew EY. Age-related macular degeneration. *Lancet* **372** (2008) 1835–1845. doi:10.1016/S0140-6736(08)61759-6.

Klein R, Meuer SM, Myers CE, Buitendijk GH, Rochtchina E, Choudhury F, et al. Harmonizing the classification of age-related macular degeneration in the three-continent AMD consortium. *Ophthalmic Epidemiology* **21** (2014) 14–23. doi:10.3109/09286586.2013.867512.

Flaxel CJ, Adelman RA, Bailey ST, Fawzi A, Lim JI, Vemulakonda GA, et al. Age-Related Macular Degeneration Preferred Practice Pattern®. *Ophthalmology* **127** (2020) P1–P65. doi:10.1016/j.ophtha.2019.09.024.

Ohno-Matsui K, Kawasaki R, Jonas JB, Cheung CM, Saw SM, Verhoeven VJ, et al. International photographic classification and grading system for myopic maculopathy. *American Journal of Ophthalmology* **159** (2015) 877–883. doi:10.1016/j.ajo.2015.01.022.

IMSS. Detección de retinopatía diabética en primer nivel de atención. Instituto Mexicano del Seguro Social (2014), 1–17 .

Bhaskaranand M, Ramachandra C, Bhat S, Cuadros J, Nittala MG, Sadda SR, et al. The value of automated diabetic retinopathy screening with the eyeart system: A study of more than 100,000 consecutive encounters from people with diabetes. *Diabetes Technology & Therapeutics* **21** (2019) 635–643. doi:10.1089/dia.2019.0164. PMID: 31335200.

Nissen TPH, Nørgaard TL, Schielke KC, Vestergaard P, Nikontovic A, Dawidowicz M, et al. Performance of a support vector machine learning tool for diagnosing diabetic retinopathy in clinical practice. *Journal of Personalized Medicine* **13** (2023). doi:10.3390/jpm13071128.

Arenas-Cavalli JT, Abarca I, Rojas-Contreras M, Bernuy F, Donoso R. Clinical validation of an artificial intelligence-based diabetic retinopathy screening tool for a national health system. *Eye (Lond)* **36** (2022) 78–85. doi:10.1038/s41433-020-01366-0.

Ting DSW, Cheung CYL, Lim G, Tan GSW, Quang ND, Gan A, et al. Development and validation of a deep learning system for diabetic retinopathy and related eye diseases using retinal images from multiethnic populations with diabetes. *Jama* **318** (2017) 2211–2223.

Ramachandra C, Bhat S, Bhaskaranand M, Nittala MG, Sadda SR, Solanki K. Advanced retinal image analysis for amd screening applications. *Investigative Ophthalmology & Visual Science* **56** (2015) 3964–3964.

Mendez-Hernandez C, Gutierrez-Diaz E, Pazos M, Gimenez-Gomez R, Pinazo-Duran MD. Agreement between five experts and the laguna onhe automatic colourimetric application interpreting the glaucomatous aspect of the optic nerve. *Journal of Clinical Medicine* **12** (2023). doi:10.3390/jcm12175485.

Yim J, Chopra R, Spitz T, Winkens J, Obika A, Kelly C, et al. Predicting conversion to wet age-related macular degeneration using deep learning. *Nature Medicine* **26** (2020) 892–899.
